# Grape seed extract attenuates arsenic-induced nephrotoxicity in rats

**DOI:** 10.3892/etm.2013.1381

**Published:** 2013-11-05

**Authors:** JIANGONG ZHANG, XINJUAN PAN, NING LI, XING LI, YONGCHAO WANG, XIAOZHUAN LIU, XINJUAN YIN, ZENGLI YU

**Affiliations:** 1 Affiliated Cancer Hospital of Zhengzhou University, Zhengzhou, Henan 450008, P.R. China; 2 Henan Cancer Hospital, Zhengzhou, Henan 450008, P.R. China; 3 School of Public Health, Zhengzhou University, Zhengzhou, Henan 450001, P.R. China; 4 College of Food Science and Technology, Henan Agriculture University, Zhengzhou, Henan 450002, P.R. China

**Keywords:** grape seed extract, chronic arsenic exposure, nephrotoxicity, oxidative stress, NADPH oxidase, TGF-β/Smad

## Abstract

Oxidative stress is a recognized factor in nephrotoxicity induced by chronic exposure to inorganic arsenic (As). Grape seed extract (GSE) possesses antioxidant properties. The present study was designed to evaluate the beneficial effects of GSE against arsenic-induced renal injury. Healthy, male Sprague-Dawley rats were exposed to As in drinking water (30 ppm) with or without GSE (100 mg/kg) for 12 months. The serum proinflammatory cytokine levels and mRNA expression levels of fibrogenic markers in the renal tissues were evaluated using enzyme-linked immunosorbent assay and quantitative polymerase chain reaction, respectively. The protein expression levels of nicotinamide adenine dinucleotide phosphate (NADPH) subunits, transforming growth factor-β1 (TGF-β1) and phosphorylated Smad2/3 (pSmad2/3) were assessed using western blot analysis. The results demonstrated that cotreatment with GSE significantly improved renal function, as demonstrated by the reductions in relative kidney weight (% of body weight) and blood urea nitrogen, and the increase in the creatinine clearance capacity. GSE attenuated the As-induced changes in the serum levels of tumor necrosis factor-α (TNF-α), interleukin-6 (IL-6) and IL-1β and the mRNA levels of TGF-β1, α-smooth muscle actin (α-SMA), connective tissue growth factor (CTGF) and fibronectin (FN) in renal tissue. Furthermore, administration of GSE markedly reduced As-stimulated reactive oxygen species (ROS) production and Nox activity, as well as the protein expression levels of the NADPH subunits (Nox2, p47phox and Nox4). In addition, GSE cotreatment was correlated with a significant reduction in TGF-β/Smad signaling, as demonstrated by the decreased protein levels of TGF-β1 and pSmad2/3 in renal tissue. This study indicated that GSE may be a useful agent for the prevention of nephrotoxicity induced by chronic exposure to As. GSE may exert its effects through the suppression of Nox and inhibition of TGF-β/Smad signaling activation.

## Introduction

Arsenic (As) is a naturally occurring element that is ubiquitously present in the environment. Chronic exposure to inorganic As has been indicated to be correlated with chronic changes in a number of organs, including the liver, kidney, skin and bladder. The kidney, as the primary organ for the excretion of metabolites, appears to be one of the main targets of As (1). The toxicity of As in the kidney has been demonstrated in the human population and animals through renal pathology and functional changes (2–4). In addition, the mechanisms of As-induced kidney damage have been investigated in numerous studies (5). Among the suggested mechanisms, oxidative stress is one of the best-accepted theories (6–8). It has been indicated that As is capable of increasing the generation of reactive oxygen species (ROS), such as intracellular peroxide, superoxide anion radical (O_2_^•−^), hydrogen peroxide (H_2_O_2_) and hydroxyl free radicals (OH^•^), which stimulate proinflammatory and profibrogenic cytokines (9) and are able to directly or indirectly damage cellular DNA and protein (7). Therefore, ROS are a significant causal factor in As-induced renal nephrotoxicity and fibrosis.

Transforming growth factor-β1 (TGF-β1) is a potent profibrogenic cytokine, and elevated TGF-β1 levels are causatively involved in the activation of profibrotic signaling pathways initiated by oxidative stress (10). The TGF-β signaling pathway is regulated predominantly by Smads. TGF-β-activated Smad pathways are pivotal for the induction of extracellular matrix (ECM) generation, myofibroblast differentiation, epithelial-mesenchymal transformation (EMT) and disease progression (11–14). Having reviewed the mechanisms of As-induced renal nephrotoxicity and fibrosis, we hypothesized that scavenging ROS, reducing oxidative stress and/or inhibiting the activities of proinflammatory and profibrogenic cytokines may partially reduce As-induced nephrotoxicity. Grape seed extract (GSE), which is rich in polyphenols, has been demonstrated to possess potent antioxidant properties and is considered to be a safe and effective antioxidant compound. It has been shown that the antioxidant activity of GSE is greater than that of vitamins C and E and β-carotene (15). GSE may exert its antioxidant and anti-inflammatory effects (16) by scavenging oxygen free radicals, inhibiting lipid peroxidation and the formation of inflammatory cytokines, altering cell membrane receptors and intracellular signaling pathway proteins, and modulating gene expression (17). In a previous study, we demonstrated that GSE may inhibit As-induced rat liver injury (18).

The initial aim of the present study was to elucidate whether dietary supplementation with GSE was capable of inhibiting chronic As-induced renal injury and fibrosis. A further aim was to explore the molecular mechanisms implicated in the action of GSE by measuring the effects of GSE treatment on nicotinamide adenine dinucleotide phosphate (NADPH) oxidase (Nox) activity and the TGF-β signaling pathway. In this study, rats received a life-long, non-lethal dosage of inorganic sodium arsenite (NaAsO_2_, As; 30 ppm in drinking water) for 12 months, with or without the co-administration of GSE and the effects on oxidative stress and TGF-β/Smad signaling were evaluated.

## Materials and methods

### Chemicals and animal treatments

The As used in the study was purchased from Sigma (St. Louis, MO, USA). GSE, containing monomeric catechins, dimeric and trimeric procyanidins, and larger procyanidins (Table I), was obtained from Jianfeng, Inc. (Lot no. G050412; Tianjin, China).

All animal procedures were performed in accordance with the Animal Care and Use Committee of Zhengzhou University (Zhengzhou, China). The experimental protocols were approved by the Animal Care and Use Committee of Zhengzhou University. The study used healthy, male Sprague-Dawley rats (6 weeks of age; average body weight bw, 180±10 g), which were randomly divided into four groups (Groups 1–4). The rats in each group (10 rats per group) were treated as follows: Group 1 received only drinking water; Group 2 received As in the drinking water at a concentration of 30 ppm; Group 3 received 100 mg/kg GSE, which was dissolved in the drinking water, every other day by oral gavage; and Group 4 received As plus GSE, with dosages and treatments as mentioned for Groups 2 and 3, respectively. The animals were housed in groups of three rats per cage at 22°C with a 12-h light/dark cycle and were given free access to food and water. The food and water intake, as well as the body weight of the animals, were monitored throughout the 12-month experimental period.

### Blood collection and tissue preparation

At the end of experiment, the rats were placed in individual metabolic cages for a 24-h urine collection. To eliminate contamination of the urine samples, the rats received only water during the collection period. Following the urine collection, the animals were immediately anesthetized with ether and the blood was collected using cardiac puncture. The blood was allowed to clot and was subsequently centrifuged and stored at −80°C for analysis. The renal tissues were collected and used for protein extraction or total RNA isolation, as well as for the analysis of ROS production or Nox activity. The protein concentrations were assessed using a protein assay kit (Bio-Rad, Hercules, CA, USA) and bovine serum albumin was used as a standard.

### Renal function parameters

Blood urea nitrogen (BUN), urinary protein (Up) levels, plasma creatinine (P_Cr_) and creatinine clearance (C_Cr_) were measured using commercial assay kits, in accordance with the manufacturer’s instructions (Bioassay Systems LLC, Hayward, CA, USA).

### Oxidative damage analysis

ROS production in the renal tissues was determined using a 2′,7′-dichlorofluorescin diacetate (DCF-DA; Invitrogen Life Technologies, Carlsbad, CA, USA) assay, where DCF-DA was converted into highly fluorescent DCF by cellular peroxides (including H_2_O_2_), as previously described (19). Nox activity in the cell membranes and cytosolic fractions was measured through the detection of ROS production using a lucigenin-derived chemiluminescence assay with NADPH as the substrate, as previously described (20). Protein carbonyls (PCs) were analyzed using the 2,4-dinitrophenylhydrazine (DNPH) method, as previously described (21). Lipid peroxidation was assessed using a thiobarbituric acid reactive substances (TBARS) assay (22). All the chemicals used were analytic grade and purchased from Sigma.

### Levels of proinflammatory cytokines

Serum levels of tumor necrosis factor-α (TNF-α), interleukin-6 (IL-6) and IL-1β were quantified using Quantikine enzyme-linked immunosorbent assay (ELISA) kits for TNF-α, rat IL-6 and rat IL-1β (R&D Systems, Minneapolis, MN, USA), respectively, in accordance with the manufacturer’s instructions.

### Quantitative polymerase chain reaction (qPCR)

Total RNA was extracted from the kidney tissue using TRIzol^®^ reagent (Gibco, Grand Island, NY, USA). cDNA was transcribed from 2 μg RNA using a high-capacity cDNA reverse transcription kit (Applied Biosystems, Foster City, CA, USA), in accordance with the manufacturer’s instructions. The target genes were amplified using Power SYBR^®^ Green PCR Master Mix reagent (Applied Biosystems). The amplification was performed in a Real-Time PCR system (Applied Biosystems 7500 systems; Applied Biosystems) and modified PCR cycles were used, as follows: Initial denaturation at 95°C for 2 min, followed by 35 cycles at 95°C for 30 sec and 60°C for 30 sec. The housekeeping gene β-actin was used as an internal control, and gene-specific mRNA expression was normalized against β-actin expression. Relative quantification using the 2^−ΔΔCT^ method was performed by comparisons with the control group. The primer sequences are summarized in Table II.

### Western blot analysis

Aliquots of the renal tissues were homogenized in ice-cold lysis buffer [1% NP-40; 10% glycerol; 20 mM Tris-Cl, pH 7.5; 150 mM NaCl; 1 mM EDTA; 1 mM ethylene glycol-O,O′-bis(2-aminoethyl)-N,N,N′,N-tetraacetic acid (EGTA), 1 mM NaVO_4_, 10 mM NaPO_4_, 10 μg/ml leupeptin and 1 mM phenylmethylsulfonyl fluoride (PMSF)]. Total protein (30–50 μg) was subjected to 12% SDS-PAGE, transferred to nitrocellulose membranes and incubated with primary antibodies against p47phox (sc-14015), Nox2 (sc-27635), Nox4 (sc-30141), β-actin (sc-8432), TGF-β1 (sc-146; all from Santa Cruz Biotechnology, Inc., Santa Cruz, CA, USA), phosphorylated Smad3 (pSmad3; cat. no. 9514) or pSmad2 (cat. no. 3101; both from Cell Signaling Technology, Inc., Danvers, MA, USA) overnight at 4°C. The membranes were subsequently probed with horseradish peroxidase-coupled secondary antibodies (Santa Cruz Biotechnology, Inc.) at room temperature for 1 h, prior to being washed again and visualized using an enhanced chemiluminescence reaction (Amersham ECL™ Western Blotting System; Amersham, Piscataway, NJ, USA). The protein expression was quantified densitometrically using LabWorks 4.5 software of American UVP Bioimaging System (Upland, CA, USA), and changes in expression were normalized to the internal standard, β-actin.

### Statistical analysis

The grouped data were evaluated using SPSS 13.0 statistical software (SPSS, Inc., Chicago, IL, USA). The methods used to test the hypothesis included one-way analysis of variance (ANOVA), followed by the Least Significant Difference (LSD) test. A value of P<0.05 was considered to indicate a statistically significant difference. The results are expressed as the mean ± standard deviation.

## Results

### GSE improves As-induced nephrotoxicity

No rats died during the 12-month experimental period. No significant differences were observed in the food and water consumption among the four groups of animals. However, As-treated rats had lower body weights and greater kidney weights and ratios of kidney weight to body weight compared with the rats in the control group. Moreover, chronic As exposure increased BUN, Up and P_Cr_ levels and decreased C_Cr_, indicating that As treatment adversely affected renal function. These changes were significantly attenuated in the rats by cotreatment with GSE (Table III).

### GSE attenuates the As-induced production of proinflammatory cytokines

Inflammation has been demonstrated to be important in the initiation of tubulointerstitial injury. To gain further insight into the mechanisms underlying the action of GSE, the expression levels of important proinflammatory cytokines known to be involved in the fibrotic process were studied. As shown in Table IV, the serum levels of IL-1β, IL-6 and TNF-α were significantly elevated in the rats in the As-treated group as compared with those in the control group (P<0.01). These effects, however, were suppressed by the simultaneous administration of GSE (P<0.01), suggesting that GSE ameliorated the As-induced hepatic inflammatory response (Table IV).

### GSE alleviates As-induced oxidative damage

To examine whether GSE modified the ROS production caused by chronic As exposure, renal tissue ROS production was assessed using DCF-DA. In addition, the renal levels of lipid peroxidation were measured according to TBARS formation, while endogenous protein oxidation was measured according to the levels of PCs. The results revealed that chronic As exposure resulted in high levels of ROS, TBARS and PC production, whereas GSE treatment significantly ameliorated these changes in As-treated rats (Table V).

### GSE attenuates As-stimulated mRNA expression of fibrogenic genes

**Figure 1:**
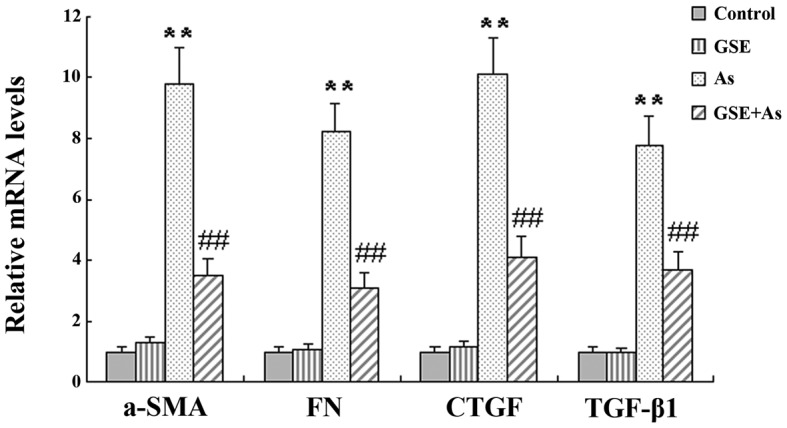
Effects of grape seed extract (GSE) on the arsenic (As)-induced intrahepatic mRNA levels of profibrogenic genes [α-smooth muscle actin (α-SMA), transforming growth factor-β1 (TGF-β1), connective tissue growth factor (CTGF) and fibronectin (FN)] were analyzed using quantitative polymerase chain reaction (qPCR) in rat livers. The mRNA levels of the control rats were arbitrarily set to 1 and all other values were calculated as multiples thereof. The transcript levels were corrected for β-actin levels. Results are presented as the mean ± standard deviation (n=10). ^**^ P<0.01 compared with control rats; ^##^ P<0.01 compared with As-treated rats.

It has been shown that chronic As exposure induces renal fibrosis. In the present study, this was corroborated by the quantification of the mRNA levels of various profibrogenic genes, specifically, α-smooth muscle actin (α-SMA), TGF-β1, connective tissue growth factor (CTGF) and fibronectin (FN). However, GSE cotreatment significantly attenuated the changes in the profibrogenic gene mRNA levels in the As-treated rat liver tissues (Fig. 1).

### GSE inhibits As-induced Nox

**Figure 2:**
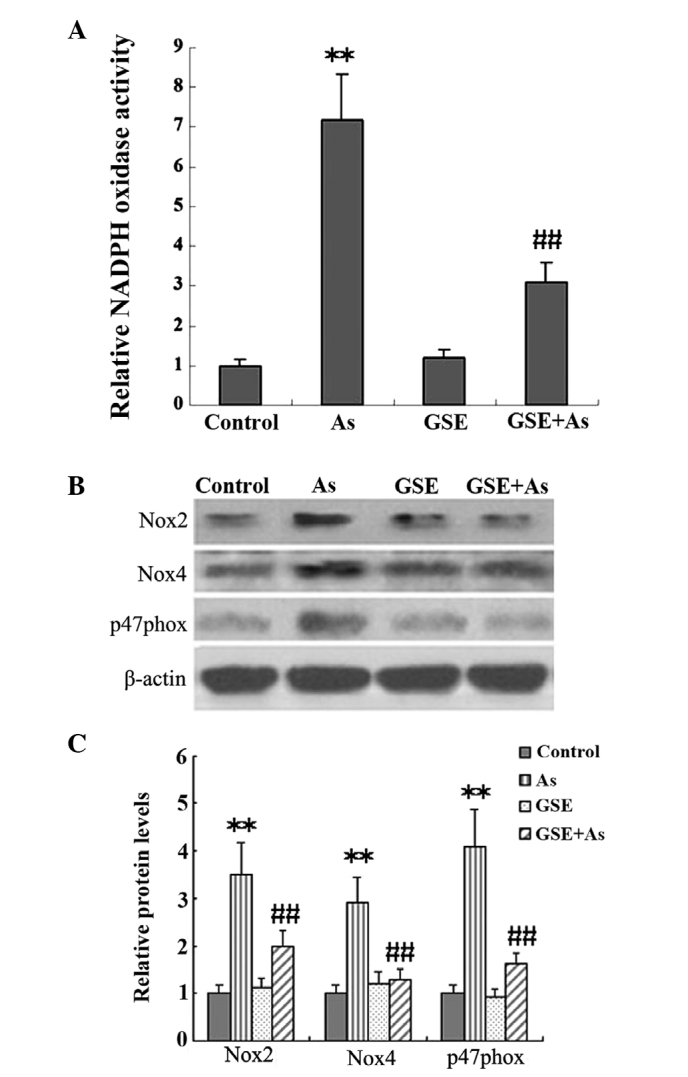
Grape seed extract (GSE) inhibits arsenic (As)-induced nicotinamide adenine dinucleotide phosphate (NADPH) oxidase (Nox). (A) Nox activity in isolated plasma membrane fractions of fresh kidney tissue homogenates was measured using chemiluminescence. Data are presented as the fold change relative to the control taken as 1.0. ^**^ P<0.01 compared with control rats; ^##^ P<0.01 compared with As-treated rats. (B) Renal tissue was homogenized and total proteins extracted, as detailed in Materials and methods. The protein levels of NADPH subunits (Nox2, p47phox and Nox4) were analyzed using western blotting. (C) Western blotting signals were quantified using densitometry and expressed in barograms as the mean ± standard deviation (n=10). Data normalized to the internal standard, β-actin, are presented as the fold change relative to the control taken as 1.0. ^**^ P<0.01 compared with control rats; ^##^ P<0.01 compared with As-treated rats.

It has been demonstrated that NADPH-derived ROS generation is pivotal in the progression of renal fibrosis. Therefore, in the present study, Nox activity and the protein expression levels of the NADPH subunits were assessed in kidney tissues, and the effects of GSE cotreatment on these variables were studied. The Nox activity and protein expression levels of the NADPH subunits, Nox2, p47phox and Nox4, were significantly elevated in the As-treated rats as compared with the controls, and these increases were significantly alleviated by GSE cotreatment (Fig. 2).

### GSE decreases As-induced TGF-β1/Smad signaling

**Figure 3:**
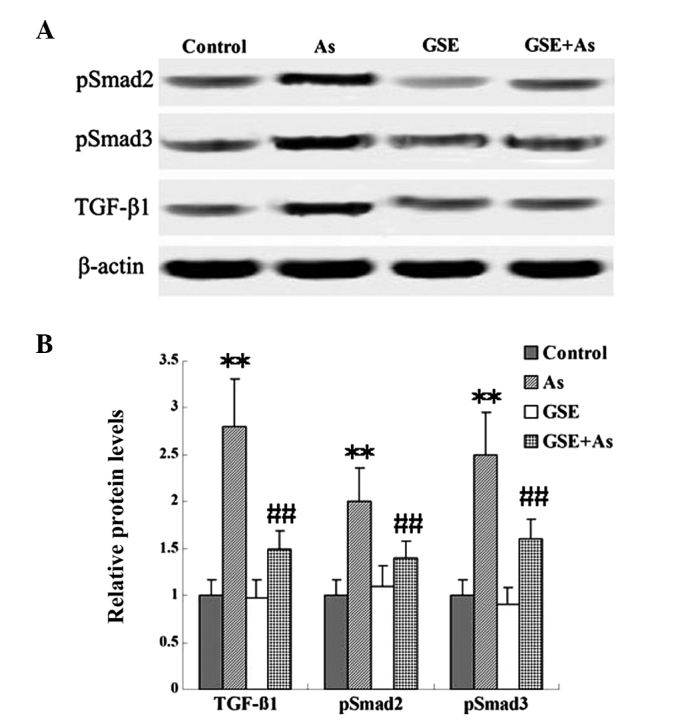
Grape seed extract (GSE) decreases arsenic (As)-induced transforming growth factor-β1 (TGF-β1)/Smad signaling. Protein levels of TGF-β1 and phosphorylated Smad2/3 (pSmad2/3) in renal tissues were evaluated using western blot analysis. (A) A total of 60 μg kidney tissue lysates were used in each SDS-PAGE lane. (B) Western blotting signals were quantified using densitometry and expressed in barograms as the mean ± standard deviation (n=10). Data normalized to the internal standard, β-actin, are presented as the fold change relative to control taken as 1.0. ^**^ P<0.01 compared with the control rats; ^##^ P<0.01 compared with As-treated rats.

TGF-β is an important signal transduction pathway mediator for renal fibrogenesis, which mediates its profibrotic effects by activating receptor-associated Smads (Smad2 and Smad3). In the present study, the protein levels of TGF-β1 and pSmad2/3 were examined in renal tissues. As demonstrated using western blotting, chronic As-administration caused marked increases in the expression of TGF-β1 and pSmad2/3 when compared with the expression levels in the control rats; however, cotreatment with GSE attenuated these changes (Fig. 3).

## Discussion

In the present study, chronic As exposure was demonstrated to lead to renal dysfunction, as demonstrated by the increased BUN and P_Cr_ and decreased C_Cr_ levels (Table III). There is a common consensus that ROS and proinflammatory and profibrogenic cytokines, which result in oxidative damage, are important in the progression of fibrosis (23). Thus, it was plausible to hypothesize that the supplementation of an antioxidant during As treatment was likely to reduce As-induced renal fibrosis (24). GSE, which is rich in catechin, epicatechin and procyanidin, possesses greater antioxidant activity than vitamins C and E, as well as β-carotene (15), and is considered to be a safe and effective antioxidant compound. The antioxidant and anti-inflammatory capacity of GSE has been previously demonstrated in other tissues (25,26). The present study has, for the first time, to the best of our knowledge, demonstrated the protective effect of GSE against nephrotoxicity in rats following chronic As exposure at a low concentration. It was shown that cotreatment with GSE significantly improved renal function, attenuated As-induced serum levels of proinflammatory cytokines and prevented kidney fibrosis. Moreover, GSE treatment was capable of reducing As-stimulated NADPH-derived ROS generation. In addition, it was revealed that GSE cotreatment led to a significant reduction in TGF-β/Smad signaling.

In the present study, a significant increase in the level of ROS production in the As treatment group indicated that oxidative damage was implicated in the nephrotoxicity caused by As (Table V). Exposure to As has been shown to lead to the increased generation of ROS (27–29). Furthermore, out of numerous pathways, Nox has been suggested to be the major source of ROS generation (30,31), as demonstrated by the increased Nox activity and protein levels of NADPH subunits, Nox2, p47phox and Nox4, in the present study. However, cotreatment with GSE led to a pronounced recovery in the As-induced oxidative injury (Fig. 2). These results were indicative of the antioxidant effect of GSE.

Chronic As exposure resulted in oxidative stress in the renal tissues. Oxidative stress may have caused further lipid peroxidation (Table V), directly damaging the membranes of cells and organelles and leading to the release of reactive aldehydes with potent proinflammatory (Table IV) and profibrotic (Fig. 1) properties. This, in turn, may have promoted oxidative stress. TGF-β1, as the most potent profibrogenic cytokine, is responsible for matrix synthesis by mesenchymal cells, such as fibroblasts, *in vitro* and during renal fibrosis (32,33). TGF-β1 accelerates renal fibrosis by a number of mechanisms. TGF-β1 stimulates fibroblasts to convert into myofibroblasts, increases the expression of α-SMA and FN and increases the synthesis of ECM components (34). Furthermore, TGF-β1 inhibits the activity of a variety of ECM-degrading enzymes, such as matrix metalloproteinases (MMPs) and plasminogen activator, thereby inhibiting the degradation of the ECM. In addition, TGF-β1 may stimulate tubular epithelial myofibroblast transdifferentiation (35,36). The TGF-β signaling pathway is regulated predominantly by Smads. TGF-β binds to a receptor on the cell surface, forming a complex of subunits known as transforming growth factor-β receptor 1 (TGFR1) and TGFR2. TGFR1 and TGFR2 activate serine/threonine kinases that subsequently mediate signaling through the Smad family of transcriptional activators (37,38). It has been demonstrated that Smad2/3 is phosphorylated by activated TGFR1 and inhibited by Smad7. Moreover, Smad7 is capable of increasing the ubiquitin-mediated degradation of TGFR1 itself, thus preventing TGF-β signal transduction. The results of the present study indicated that the As-induced rat renal injury was correlated with TGF-β1-induced fibrosis, as demonstrated by the increased levels of TGF-β1 and pSmad2/3 in renal tissue. GSE cotreatment attenuated these changes (Fig. 3).

Based on the results of the present study, it is hypothesized that the enhanced production of ROS and proinflammatory and profibrogenic cytokines may have been the possible mechanisms underlying the As-induced oxidative stress, which was critical in the development and progression of renal fibrosis. In addition, the results demonstrate that the activation of TGF-β/Smad signaling was correlated with As-induced renal fibrosis, and that the suppression of TGF-β/Smad activation was involved in the beneficial effects of GSE. In conclusion, GSE, a powerful antioxidant with diverse beneficial effects, may be a promising agent for the prevention of renal fibrosis and dysfunction caused by chronic As exposure at a low concentration in drinking water.

## Figures and Tables

**Table I:** Composition of grape seed extract (GSE).

Compounds	% (wt/wt)
Monomers	3.7–3.9
Catechin	1.3–1.9
Epicatechin	1.4–2.0
Epicatechin gallate	1.0–1.7
Dimers + trimers	28–31
Procyanidin B1	6.8–7.4
Procyanidin B2	4.9
Procyanidin B3	3.2–3.7
Procyanidin B4	2.1
Total procyanidins	>96

Composition values based on the Vitaflavan specification (Vitaflavan French grape seed extract: polyphenols >96% ) and Lot Certificates of Analysis provided by the supplier (Jianfeng, Inc., Tianjin, China).

**Table II:** Primer sequences for quantitative polymerase chain reaction.

Gene	Forward	Reverse
TGF-β1	5′-ATACGCCTGAGTGGCTGTCT-3′	5′-TGGGACTGATCCCATTGATT-3′
α-SMA	5′-CCGAGATCTCACCGACTACC-3′	5′-TCCAGAGCGACATAGCACAG-3′
FN	5′-TGCAATGATCAGGACACCAGG-3	5′-GTAATTCCGGTTGCTGTACAG-3′
CTGF	5′-GAGCTTTCTGGCTGCACC-3′	5′-TCTCCGTACATCTTCCTG-3′
β-actin	5′-CCATTGAACACGGCATTGTC-3′	5′-TCATAGATGGGCACACAGTG-3′

TGF-β1, transforming growth factor-β1; α-SMA, α-smooth muscle actin; FN, fibronectin; CTGF, connective tissue growth factor.

**Table III:** GSE improves As-induced renal injury.

Parameter	Control group	GSE group	As group	GSE + As group
BW (g)	571.4±30.1	582.9±39.1	411.3±19.2^a^	589.3±29.7^b^
KW (g)	4.19±0.69	4.23±1.01	5.01±0.87	4.55±0.99
KW/BW (g/kg)	7.33±1.6	7.26±1.2	12.10±1.7^a^	7.72±1.5^b^
Up (mg/day/100 g BW)	20.1±1.6	18.3±2.4	45.3±9.1^a^	29.6±7.3^b^^a^
P_Cr_ (mg/dl)	0.68±0.09	0.67±0.08	1.90±0.15^a^	1.10±0.11^b^
BUN (mg/dl)	31.1±4.6	30.8±4.9	58.9±11.2^a^	40.1±9.7^b^
C_Cr_ (ml/min)	4.8±0.7	4.9±0.9	2.9±0.1^a^	3.9±0.4^b^

Results are presented as the mean ± standard deviation (n=10).

a Values differ significantly from those of control rats (P<0.01);

b values differ significantly from those of arsenic (As)-treated rats (P<0.01).

GSE, grape seed extract; BW, body weight; KW, kidney weight; Up, urinary protein; P_Cr_, plasma creatinine, C_Cr_, creatinine clearance; BUN, blood urea nitrogen.

**Table IV:** Serum proinflammatory cytokine levels in different treatment groups.

Cytokine	Control group	GSE group	As group	GSE + As group
IL-1β (pg/ml)	17.1±6.1	15.3±5.7	58.3±17.1^a^	22.9±7.2^b^
IL-6 (pg/ml)	33.5±9.6	34.1±6.5	73.2±10.1^a^	38.9±8.8^b^
TNF-α (pg/ml)	13.9±3.3	11.9±2.3	37.7±10.1^a^	16.4±9.1^b^

Results are presented as the mean ± standard deviation (n=10).

a Values differ significantly from those of control rats (P<0.01);

b values differ significantly from those of arsenic (As)-treated rats (P<0.01).

GSE, grape seed extract; IL, interleukin; TNF-α, tumor necrosis factor-α.

**Table V:** Levels of oxidative damage in the rat kidneys in different treatment groups.

Oxidative stress marker	Control group	GSE group	As group	GSE + As group
ROS (pmol/mg protein)	4.5±0.99	4.3±1.02	13.7±1.00^a^	5.9±1.33^b^
TBARS (nmol/mg protein)	0.33±0.07	0.29±0.08	0.71±0.09^a^	0.46±0.07^b^
PCs (nmol/mg protein)	1.66±0.36	1.71±0.19	4.50±0.45^a^	2.20±0.33^b^

Results are presented as the mean ± standard deviation (n=10).

a Values differ significantly from those of control rats (P<0.01);

b values differ significantly from those of arsenic (As)-treated rats (P<0.01).

GSE, grape seed extract; ROS, reactive oxygen species; TBARS, thiobarbituric acid reactive substances; PCs, protein carbonyls.

## Abbreviations

NADPH: nicotinamide adenine dinucleotide phosphate
BUN: blood urea nitrogen
C_Cr_: creatinine clearance
P_Cr_: plasma creatinine
α-SMA: α-smooth muscle actin
FN: fibronectin
CTGF: connective tissue growth factor
GSE: grape seed extract
ROS: reactive oxygen species
Nox: NADPH oxidase
PCs: protein carbonyls
TBARS: thiobarbituric acid reactive substances
